# What training and support do people need to make optimal use of automated insulin delivery systems: Interview study

**DOI:** 10.1111/dme.70153

**Published:** 2025-10-03

**Authors:** Julia Lawton, Catriona J. Kyle, Thomas J. G. Chambers, Lesley Steven, Ashton Currie, Fraser Gibb, John A. McKnight, David Rankin

**Affiliations:** ^1^ Usher Institute, Medical School University of Edinburgh Edinburgh UK; ^2^ Usher Institute, NHS Lothian Western General Hospital Edinburgh UK; ^3^ Centre for Discovery Brain Science University of Edinburgh Edinburgh UK; ^4^ NHS Lothian, Royal Infirmary of Edinburgh Edinburgh UK

**Keywords:** automated insulin delivery (AID) system, hybrid closed‐loop, quality‐of‐life, training and support, type 1 diabetes, users' perspectives

## Abstract

**Introduction:**

Despite increased use of automated insulin delivery (AID) systems, limited attention has been paid to users' training and support needs. Indeed, most guidance has been driven by clinician opinion rather than users' experiences and needs. We explored the experiences of adults with type 1 diabetes initiated onto AID to make recommendations for supporting optimal use of AID systems in routine clinical practice.

**Methods:**

We conducted interviews with *n* = 22 individuals who used the Tandem t:slim X2™ with Control‐IQ technology AID system for ≥6 and ≤12 months. Data were analysed thematically.

**Results:**

Users reported wide‐ranging clinical and quality‐of‐life benefits. However, to actualise these benefits, they highlighted a need for bespoke training which took account of their learning preferences and strengths, pace of learning and prior insulin pump experience, together with ongoing support from healthcare professionals. Thematic analysis uncovered four overlapping support needs: (1) support reviewing data and making adjustments to the system's settings, (2) emotional/psychological support to help sustain motivation and/or adopt more passive self‐management roles, (3) support to unlearn unhelpful behaviours and/or establish new habits and routines; and (4) educational support.

**Conclusions:**

AID systems are an exciting advance in diabetes treatment. However, to achieve optimal gains, users would benefit from initial training and ongoing glycaemic, educational, and psychosocial support tailored to their preferred ways of learning and personal circumstances. Consideration should be given to adapting existing education and training packages for AID system users to incorporate behavioural science techniques and upskilling diabetes professionals in behaviour change approaches and motivational interviewing.

**What's new?**

- Use of automated insulin delivery (AID) systems is escalating, but best practice guidelines have largely been informed by clinician opinion rather than users' perspectives, experiences and support needs.
- To gain maximum benefit from this technology, our interviews with AID system users highlighted a need for initial training tailored to different learning preferences and strengths, and bespoke follow‐up support comprising behavioural, psychological, clinical and educational components.
- Consideration should be given to adapting existing education and training packages for AID system users to incorporate behavioural science techniques and upskilling diabetes professionals in behaviour change and motivational interviewing.

## 1 INTRODUCTION

Automated insulin delivery (AID) systems (also known as hybrid closed‐loop systems) link an insulin pump with a glucose sensor via an algorithm to help automate insulin delivery. While slightly different in terms of their (algorithmic) functionality, all commercially available systems require some user input (e.g., announcing carbohydrate intake), although the type of input required varies between systems.^1^ AID systems have been widely investigated in clinical trials involving people with type 1 diabetes (T1D) and shown to lead to improvements in glycaemic outcomes^2^ and quality‐of‐life, albeit the evidence for the latter is more equivocal.^2^, ^3^

Various systems have now been commercially approved and their use is growing rapidly. In the United Kingdom, a recent landmark moment was the decision to make AID systems freely available to all adults with T1D who have an HbA1c >58 mmol/mol (7.5%) or disabling hypoglycaemia, despite best attempts to manage glycaemia using an insulin pump and/or glucose sensor.^4^ Similar developments have been reported in other countries.^5^ The escalation of AID system use is exciting but is also likely to present significant challenges because healthcare professionals are unlikely to be able to offer the same level of input and support as they have typically provided to participants in clinical trials.^6^, ^7^ Indeed, when ‘real world’ research has been undertaken, there are some indications that there is a higher risk of dropout than in clinical trials.^6^, ^8^, ^9^, ^10^ In addition, both clinical trial and ‘real world’ research have shown that while most users experience significant glycaemic improvements, many do not attain optimal (≥70%) time‐in‐range^10^, ^11^; with some qualitative research further indicating that some individuals do not always use these systems as clinically advised.^6^, ^12^, ^13^, ^14^ Hence, further research is needed to develop evidence‐based interventions which can be used to support the use of AID systems in routine clinical care.^5^

While a plethora of studies have explored people's lived experiences of using AID systems, these have overwhelmingly focused on perceived benefits/burdens and how system use affects people's diabetes self‐management and everyday lives.^15^, ^16^, ^17^, ^18^, ^19^, ^20^ Very limited attention has been paid to users' training and support requirements, which might help explain why best practice guidelines and consensus statements have mostly been informed by clinician opinions and expertise^21^, ^22^, ^23^, ^24^ rather than users' perspectives and experiences.

In this article, we report findings from interviews with a demographically mixed group of adults living with T1D who used the Control‐IQ AID system. We explored users' experiences, training, and support needs to make recommendations to support optimal system use in routine clinical care.

## 2 METHODS

### 2.1 Overview

Semi‐structured interviews were chosen for this study to allow us to capture the information needed to address the study objectives, while affording flexibility for interviewees to raise issues they considered salient, including those unforeseen at the study outset. We undertook data collection and analysis concurrently to enable issues identified in early interviews to inform areas explored in later ones. Given the applied nature of this study, and our commitment to providing practical and tangible recommendations, our methodological orientation was informed by a qualitative descriptive approach,^25^ which is a pragmatic approach involving the identification and description of minimally theorised explanatory themes.

We interviewed adults with T1D who used the Tandem t:slim X2™ with Control‐IQ technology AID system during a single‐centre observational study based in Scotland.^26^ Participants, who were existing t:slim X2 pump users, were recruited into the observational study from existing National Health Service (NHS) waiting lists for continuous glucose monitoring (CGM), with no exclusion criteria applied. Mirroring provisioning within the NHS, participants had to complete mandatory online training modules and attend a group‐based training session delivered by an Air Liquide representative before commencing system use.

Further details about the observational study and the Control‐IQ AID system used are provided in Box 1. Additional information about the training given to participants and research staff is provided in Box 2. Ethics and governance approvals were obtained as part of the main observational study (REC ref.: 21/NS/0134).

BOX 1 **Description of the observational study and Control‐IQ AID system**

**Table:** 

*The main study* The observational study was conducted at a single clinical site in Scotland and recruited 30 adults from diabetes and insulin pump clinics. To be eligible, participants had to: be aged ≥18 years; have T1D; have been established on the Tandem t:slim X2 pump for ≥1 month; and, have an HbA1c of 40–90 mmol/mol (5.8%–10.4%). People who were pregnant or planning pregnancy were excluded. *The Tandem Control‐IQ AID system* The Tandem t:slim X2™ with Control‐IQ technology AID system comprises the Tandem t:slim X2™ insulin pump (Tandem Diabetes Care, San Diego, CA, USA) which communicates wirelessly to the Dexcom G6 CGM (Dexcom, San Diego, CA, USA). The Control‐IQ algorithm (hosted on the pump) uses CGM and delivered insulin data to predict glucose levels 30 min ahead, and if required, make basal insulin adjustments every 5 min. When predicted glucose values are within target range, the pump delivers insulin based on the active personal profile settings (manually set basal rates and correction factors). Insulin duration is set at 5 h. Usually, the algorithm aims to maintain glucose levels between 6.3–8.9 mmol/L. If sensor glucose levels are predicted to exceed 8.9 mmol/L, basal insulin is increased. If sensor glucose is predicted to exceed 10.0 mmol/L the system delivers an automatic correction bolus (one per hour) with a target of 6.1 mmol/L. If glucose values are predicted to fall below 6.3 mmol/L, basal insulin is decreased then suspended at 3.9 mmol/L, resuming progressively above this value. The system includes further functions enabling users to: Use a sleep activity mode, which can be manually set or pre‐programmed, where the algorithm aims to maintain glucose levels between 6.3–6.7 mmol/L. Automatic corrections are suspended during sleep. Programme and customise an exercise mode where the algorithm aims to maintain glucose levels between 7.9–8.9 mmol/L. Automatic corrections continue to be administered when using the exercise function. Set (up to six) personal profiles to define basal rate, correction factor and ICRs to facilitate insulin delivery within specific time segments over a 24‐h period. Set an extended bolus to alter duration of bolus insulin delivery over a period of up to 8‐h. View summary data (statistics) via Glooko and Clarity downloads, including time‐in‐range, time‐above‐range, time‐below‐range, estimated HbA1c, average glucose level and total daily dose of insulin. Participants also had access to their ambulatory glucose profile to view trends in their glucose levels. Receive and personalise pump, CGM and Control‐IQ alerts (audio and vibrations via the pump), for example, triggered by predicted high/low glucose levels or when pump and CGM are out‐of‐range.

BOX 2 **Training received by study participants and study staff.**

**Table:** 

*Training and support provided to participants* In accordance with routine clinical practice, all participants had to complete mandatory online modules (interactive slides), including the Dexcom G6 Sensor Sessions and Control‐IQ Technology Module on the t:slim X2 insulin pump. This module provided information on: how Control‐IQ technology works; status indicators to view what Control‐IQ is doing; set‐up procedures; activity settings for sleep and exercise and responses to Control‐IQ alerts (e.g., for low/high glucose levels); and, concluded with a quiz which participants were required to pass. Participants then attended a (1 hr) group‐based switch‐on session involving up to 12 people from a range of countries with varying degrees of experience of using an insulin pump, delivered via an online platform by an Air Liquide representative. During this session participants received: instruction about how to initiate Control‐IQ (entering weight, total daily dose and sensor code); information about setting and using sleep and exercise modes; advice about setting different personal profiles including inputting basal rates, correction factors and ICR; advice about and responses to low/high glucose alerts; setting/using the extended bolus feature; operating parameters (range) of the system and tips to maintain connection between components; information about data (e.g., TIR) available on the system; and, opportunities to ask questions. Participants were also provided with written information, including: the t:slim X2 Insulin Pump with Control‐IQ Technology User Guide, a Dexcom G6 CGM system manual, and information about uploading to the Dexcom Clarity system and Glooko. After commencing use of the Control‐IQ system, participants attended at 4, 12, 26, 52 weeks to meet with study staff (research nurses) to provide blood samples, information about hypoglycaemia, complete questionnaires, and download/review pump and CGM data. At these visits, participants were invited to raise concerns (e.g., regarding hypo/hyperglycaemia, nocturnal hypos or exercise/lifestyle issues) with members of the research team who would review and discuss data and then, if necessary, advise about changes to basal rates, ICRs and correction factors. Participants were also advised that they could contact the research team to arrange additional (unscheduled) visits or seek input by phone/email if any issues or concerns arose before their next scheduled visit. *Study‐related training received by members of the research team* Prior to the study beginning, members of the research team attended preparation meetings involving representatives from Air Liquide to ensure that procedures and processes were in place to access and review participants' data, and attended a switch‐on session to observe the training received by participants. Research staff attended the Air Liquide Healthcare Academy training session prior to the study commencing and a similar session the following year. The study team also received technical and trouble‐shooting support from representatives of Air Liquide, Dexcom and Glooko.

### 2.2 Recruitment

Participants were recruited into the interview study using an opt‐in procedure after they had ≥6 and ≤12 months' experience using the Control‐IQ system. Purposive sampling was used to seek diversity with respect to age, gender, socio‐economic status, and ethnic background. Recruitment continued until data saturation was reached.

### 2.3 Data collection

Participants were interviewed once by a highly experienced, independent (non‐clinical) qualitative researcher (DR) between June 2024 and January 2025. Interviews were informed by a topic guide (see Box 3) developed in light of literature reviews^15^, ^27^ consultation work with individuals with lived experience of using AID systems, and input from clinical co‐investigators, with further revisions made in response to emergent findings, in line with the inductive approach chosen for this study. Interviews lasting 1–2 hrs were conducted by telephone or using an online platform and were digitally recorded and transcribed verbatim.

BOX 3 **Topics explored in interviews.**

**Table:** 

*Background information and pre‐study experiences of managing diabetes* Age, diabetes duration, occupation, marital status, dependents, living arrangements, and hobbies/interests. Preferred ways of learning and preferences for learning about new technology (e.g., group, 1:1, in‐person, online, video, manual, etc.). Perceived personality type (e.g., controlling, perfectionist, laid back, etc.); (any) impact of one's personality on one's approach to diabetes self‐management. Previous experiences of managing diabetes using a pump Likes/dislikes, benefits, and challenges, Perceived impact of pump use on glucose levels; diabetes self‐management and quality‐of‐life. *Training received and experiences of using Control‐IQ* Likes/dislikes of the training received, including online modules, group‐based switch‐on training and user manual (provided by Air Liquide); perceived impact of training on knowledge and understanding of Control‐IQ and confidence and ability to use the system. Views about how the training could be improved to enhance engagement, confidence, knowledge, and understanding. Initial experiences of switching on and using the system, any difficulties encountered and why? How long did it take to get used to using the system; what helped/hindered this process? Experiences of seeking and receiving support from study staff (e.g., to make adjustments to settings such as correction factors, basal rates, insulin‐to‐carbohydrate ratios; managing low/high glucose levels; sleep; physical activity). Views about support received from study staff (what was helpful/unhelpful); any unmet needs for support; any changes in the support needed over time. Perceived impact of using the Control‐IQ system on glucose management and everyday life, including: Dietary choices and adjustments at mealtimes Relationships with other people in the household, family and friends, at work Sleep (duration, quality, nocturnal hypoglycaemia) Ability to undertake physical and other activities Life choices more generally, plans for the future Thoughts and concerns about having diabetes and diabetes complications Quality‐of‐life more generally, for example, mood, confidence, self‐esteem. *Support needs in routine clinical care* Views about what it would have been like to use the system in routine care, for example, without having access to study staff? Views about the kinds of support needed by people using the AID system in routine care, including: When they are provided with initial training and onboarding? When they are adapting to using the system and over time? Frequency of support and who should provide it?

### 2.4 Data analysis

Interviews were analysed by JL and DR using descriptive and thematic approaches informed by the method of constant comparison.^28^ Data analyses, which involved both inductive and deductive thematic approaches, involved the researchers undertaking close and repeated reading of transcripts to independently identify key areas of interest and preliminary cross‐cutting themes. Each researcher produced a separate report before meeting to discuss their interpretations and agree upon a coding framework that captured key themes and contextual information needed to aid data interpretation. The qualitative software package NVivo 14 (QSR international, Doncaster, Australia) was used to facilitate data coding and retrieval. Coded datasets were subjected to further comparative analyses to refine themes, develop subthemes, and identify illustrative quotations.

## 3 RESULTS

The final sample comprised 22 individuals—see Table 1 for more information. Below, we begin by reporting the glycaemic and quality‐of‐life benefits participants reported experiencing before considering their views about the training and support needed to actualise these benefits in everyday life. We conclude by describing participants' views about how AID system users' support needs could be met within routine clinical care. Key illustrative quotations are included below; for additional quotes see Table 2. Note, we have retained participants' use of the phrase ‘closed‐loop’ in our reporting.

**TABLE 1:** Sample characteristics, *n* = 22 adults with T1D.

Characteristic	*n*	%^a^	Mean, SD, (range)
Female	12	54.5	
Male	10	45.5	
Age at time of interview (years)			36.3 ± 8.6 (31–62)
Diabetes duration (years since diagnosis)			29.6 ± 10.2 (10–43)
Baseline HbA1c			
mmol/mol			60.1 ± 12.1 (43–89)
%			7.7 ± 1.1 (6.1–10.3)
Total duration of insulin pump use across all devices (years)			7.7 ± 2.9 (2–12)
Duration of Tandem t:slim X2 Insulin Pump use pre‐study (years)			2.1 ± 1.·3 (0.5–6)
Married/co‐habiting	19	86.4	
Employed	19	86.4	
Unemployed/student	2	9.1	
Retired	1	4.5	
Occupation^b^			
Managers	2	9.1	
Professionals	5	22.7	
Technicians and Associate Professionals	4	18.2	
Clerical Support Workers	3	13.6	
Service and Sales Workers	4	18.2	
Craft and Related Trades Workers	1	4.5	
Student/unemployed	2	9.1	
Retired	1	4.5	
Ethnicity			
White, British	18	81.8	
Polish	2	9.1	
White, other nationality	1	4.5	
Asian, British	1	4.5	

^a^ Figures may not add up to 100% due to rounding.

^b^ Defined using the International Standard Classification of Occupations 2008 (ISCO‐08).

**TABLE 2:** Supplementary quotations.

THEMES	Subthemes and additional illustrative quotations
Clinical and quality‐of‐life benefits—changed lives and selves	*Improvements in sleep* ‘I was having loads of hypos during the night…I was always waking up…treating things during the night. I've not had one hypo…since I've been on the closed loop. I feel like I can go to my bed now, just far more relaxed because if you're dipping again, it'll just stop you going too low, and you look at my bloods during the night, and they're just like a total flat line’. (022) *Advancing careers* ‘Certainly work‐wise it's freed me up with the activity and maybe not taking so many lows. In terms of my work, I'm able to do more through the day rather than take time out to deal with sugars. So, certainly from someone with their own business, being able to get my jobs done a little bit faster, better, that really has improved my life in terms of how my work week goes’. (008) *Focusing more on family life* ‘I feel more confident being with [names child] myself now…like when he was young, I just worried that what if I did have a hypo? Like what would happen to him, what would happen to me? So now I feel like I can just do things with him…I've got that safety net now’. (022)
Actualising the benefits of the AID system	***Initial training*** *Taking account of previous experience using diabetes technologies* ‘My recollection of the online training was that there were one or two people who maybe weren't as confident with technology as other folk, who maybe weren't as experienced with pumps. Given that these things are generally time constricted…I think the proportion of time they needed [and] like the tutor needs to attend to some of those issues, it kind of imbalances things, and it's maybe not the best way to do stuff’. (014)
Ongoing tailored support	***Glycaemic support*** *Glycaemic input from HCPs was pivotal to success* ‘Their help has been huge, absolutely huge, even just knowing they were there as well…they [appointments] were fairly frequent in the first instance, and then there was also telephone calls in between, just to monitor my uploads. So that helped, and then [research staff member's name] suggested a couple of things, because she's got more of an overview…so she suggested a couple of things to try and I've taken on them on board and they've worked’ (007) *Difficulties interpreting insulin and glucose data* ‘I'm not very good at looking through all the data, and like analysing it, because I think if I started doing that I'd go down a massive rabbit hole… So it's helpful to get a second pair of eyes… that actually understands what to look for’. (011) *Benefits of receiving input from staff aware of participants' personal circumstances* ‘…it's that it's personalised, that it's tailored to you. They're looking at what has actually happened to you for the last while, ‘cause not everybody reacts in the same way to things. So, if you were just getting a text book saying: if this happens, try this…well you wouldn't trust in it anywhere near as much, when it's an expert who's sat there, someone who spent time getting to know you and spent half an hour looking at your results and says: why don't you try this?’ Then yeah, you trust what they've said, and give it a shot, and yeah, they're normally right’. (017) *Support with more granular interpretations of data and recommendations* ‘I am giving a bolus now, but a very small one. [T]his is after our conversation with [research staff member's name], because there have been some scenarios where I've been giving myself carbs, but not insulin. And that would confuse the data basically because, even though it is maybe just a small amount of carbs, it will still affect my closed‐loop system… So now, even if I just have something small to eat that's maybe five grammes of carbohydrate for example, I will give myself a small bolus’. (013) ***Educational support*** *Support to use the system when doing physical activity* ‘I think they caught on to this quite quickly. Because we had a coffee morning for everyone that was on the study…because pretty much everyone had mentioned the struggles they'd had with exercise on the closed‐loop system…the margin of error can be so small if you want it to be, it is good to understand how to use the…closed‐loop system with exercise, even if you're just going for a walk, even if you are…doing some gardening or like something small’. (021)
Using AID in routine clinical care	*Risks of system discontinuation* ‘It would be more difficult [without the input of study staff]. You may see your dropout rate increasing significantly. You might see people saying they want to come off it quicker or just giving up on it and not using it. It may be used incorrectly; people may experience hypos and type in more [carbohydrate] than they should have’. (018) *Disadvantages faced by less technically/numerically literate individuals* ‘…a barrier to using the closed‐loop is…someone say elderly, or somebody who may be young and…doesn't have their parents maybe to help them, it could be quite challenging, if you didn't have…the capacity or you couldn't interpret all the results. And…you either wouldn't want the closed‐loop, because it would be too daunting and too complicated, or you might have it and you wouldn't be using it to its potential at all’. (001) *Remote data reviews* ‘even if it's a 5 minute phone‐call just to make a review…you don't have to be making the trek down to the hospital for 10 minutes when it could just be done over the phone’. (014) *A triage system to prioritise those needing support* ‘if they looked at, say monthly or fortnightly, like had a review of uploaded data, how it's going… Look[ing] to see if there are people who are particularly, after a period of time, not settling down. Looking to be more proactive about understanding why and what adjustments would help’. (019) *Offering structured education to support individuals to…* *…adjust settings*: ‘if there's new people coming onto it, I think this should be something similar to that DAFNE… Like having an education course where you're like: instead of doing those micro adjustments, you could have done this. He could have changed this profile. Maybe that would be helpful’. (001) *…refine carbohydrate counting*: ‘I think it's something that really is worthwhile to any diabetic to have the DAFNE…I think really you do need to be carb counting quite well to get the most out of the pump [closed‐loop]. You can use a pump and carb count loosely but to really get those tight sugars, where you're not worrying, I think carb counting goes a long way’. (008) *…give/receive emotional support*: ‘I wonder if there's a case for more peer support as well… like the DAFNE course that I went on years ago, and I'm still in touch with a couple of people that I got to know through that. And we do discuss things every now and then, and I guess there's somebody just to have a bit of a whinge at every now and then as well. Maybe…even if it was that level of support, you know: I'm finding this really tough now. And somebody goes: well so was I, but keep at it’. (017)

### 3.1 Clinical and quality‐of‐life benefits—changed lives and selves

Participants reported wide‐ranging clinical and quality‐of‐life benefits, with many highlighting ways in which system use had shifted their relationship with their diabetes and allowed them to gain or reclaim parts of their lives which their diabetes had taken away. A key benefit cohered around improved glucose management, with many reporting better sleep (Table 2) alongside more general improvements in their energy, mood, and sense of well‐being:

I feel better. I've got more motivation…Sometimes I felt quite anxious or even, like, depressed. I don't feel like that anymore, it's a big change. (015)

Participants also described experiencing reduced mental and physical workloads by virtue of being able to delegate glucose management tasks to the system with 003, for instance, noting how: ‘it just allows you a little bit more brain space be you, instead of being a diabetic’.

Indeed, by virtue of experiencing these benefits, together with the reassurance gained from ‘having that safety net…so your bloods can't plummet the way they did with the pump’ (022), participants described feeling more confident and able to take on roles and activities which gave them a sense of meaning and purpose. This included advancing their careers (Table 2), devoting greater time to family life (Table 2), or taking on new hobbies, such as playing football or baking, with participants suggesting that: ‘the [closed‐loop] has given me back part of my life which my diabetes had taken away’ (007).

### 3.2 Actualising the benefits of the AID system

#### 3.2.1 Initial training

To actualise the system's benefits, participants emphasised the importance of receiving initial training because, ‘it's not enough to just hand someone a device and say good luck’ (001). When they reflected upon training provided by the system manufacturer, most indicated that it had given them ‘a good overview of how this closed‐loop system works and…some tips for what to expect from using the system’ (015). However, virtually all highlighted areas for improvement.

Many, for instance, noted that the training had not accommodated, or capitalised upon, their learning preferences and strengths, such as 012 who described themselves as ‘more of a visual learner, so watching videos, being shown how to work something, works better for me’ and 016 who reported ‘hat[ing] instructions…I much prefer having a face‐to‐face relationship with someone who can show you’ (016). These individuals described the initial online modules as having been ‘boring, reading for an hour off the screen what you should and shouldn't do…as soon as I'd finished, I'd forgotten it’ (021), with many also expressing a preference for in‐person learning because this would have allowed them to: ‘physically sit with somebody, ask any questions whilst actually having your hands on the system’ (002).

Participants also suggested that the training had been too standardised and generic: ‘it felt very process…just to do this, this and this and this will happen’ (007) and expressed a preference for bespoke approaches tailored to their own pace of learning. Specifically, while some suggested that their learning had been compromised by receiving training alongside individuals who picked up information more slowly than themselves or were pump novices (Table 2), others described having felt ‘overwhelmed’ because ‘you've not used it, it's sort of something completely new and it's a very short period of time’ (009).

To further enhance their learning, many also expressed a strong preference for small group or one‐to‐one approaches as they had felt restricted in their ability to ask questions and personalise their learning due to the large number of people receiving training at the same time as themselves (typically 10–12):

I would do smaller groups…I think the idea that the more you can personalise this kind of situation the more effective it is. I think the bigger the groups I just think logically, exponentially you kind of get less value through it. (019)

#### 3.2.2 Ongoing tailored support

Despite receiving training, many described their initial days and weeks of system use as having been a ‘rude awakening’ (006), wherein:

It's not like I've woken up one morning and gone: ‘oh my god this is life changing’. But, with a lot of grunt work, I think just incrementally, tiny little bits have been lifted slowly but surely. (003)

Indeed, most participants described a long journey before they began to realise fully the system's benefits. In doing so, participants alluded to four (often) overlapping support needs; these are considered below.

##### Glycaemic support

Participants were advised to export their existing profile settings (basal rates, insulin sensitivity/correction factor) when they first transitioned onto the Control‐IQ system. After having done this, a small minority, typically those commencing the study with lower (≤50 mmol/mol) HbA1cs, described having only needed to make minor, if any, adjustments before experiencing a clinically advised time‐in‐range. The majority, however, reported an intensive and often lengthy calibration period during which study staff had needed to make multiple adjustments to their basal infusion rates, correction factors, and/or insulin‐to‐carbohydrate ratios (ICR) before the system began to perform optimally:

So when I transferred all my stuff over if anything my control got worse…And I'd assumed it was teething issues…but…they said: no…there was lots wrong like across the board…your ratio should be more like this, the bedtime should be more here, your correction dose should be this…They made a lot of tweaks. (021)

Indeed, many participants described the glycaemic input received from study staff, both during scheduled appointments and via phone/email, as having been pivotal to successful system use (Table 2), with some further noting how they would have struggled to make the necessary adjustments themselves due to their difficulties interpreting their own insulin and glucose data (Table 2).

As well as benefiting from regular data reviews, participants highlighted the importance of receiving input from study staff who had had opportunities to get to know them and learn about their personal circumstances (Table 2). As they observed, this had helped staff to make more granular interpretations of their data and recommendations for fine‐tuning their behaviours (e.g., changing the timing of pre‐meal boluses or administering insulin even for very small snacks) to help create the conditions that allowed the system to operate optimally (Table 2).

##### Emotional/psychological support

Many participants also described their initial experiences of AID system use as having been emotionally challenging and ‘really draining’ (010). Some, for instance, reported how, in the initial days and weeks, disruptions to their work or sleep schedules due to frequent and unexpected alarms had led them to question whether they would be able to persist with system use:

I was constantly battling lows, you know, like I would have hypo after hypo …I think there was one day in particular where I had 35 alarms. And I was ready to put it in the bin. (019)

Such participants reported benefitting from a cheerleading approach from study staff, especially when they felt their motivation had started to wane:

Having that sort of championing, ‘you're doing great…look how much your HbA1c has started to improve’, I think that's something that has definitely been beneficial…helping to keep the momentum when you get tired and put off. (019)

Others, especially those who described themselves as ‘lik[ing] being in control’ (021) shared their difficulties allowing their glucose to rise or fall without intervening:

It's been very much an eye‐opening experience as to how much of a control freak I am…I really did fight the machine for a good couple of months because it was doing things and it wasn't me that was actioning them…And I didn't like that. (006)

Those who reported having competitive or perfectionist traits also highlighted additional (ongoing) challenges relinquishing glucose management to the system due to wanting to attain glucose levels within a very tight time‐in‐range:

I think this is probably vanity, that I make adjustments cause I'm a little bit obsessed with looking at my percentage time‐in‐target. And maybe, yeah a little bit of vanity to try and keep it down rather than just letting the pump do its thing. (001)

Such participants reported benefiting from ‘encouragement from the research nurses, telling me to trust it and it will work’ (012). 016, for instance, who described themselves as being someone ‘who will study as much as humanly possible to make sure the chance of failure is as small as possible’ reported finding it helpful attending appointments where:

we talked about control, giving control to something that keeps you alive…[because] it took a bit of time to persuade me to stop thinking, oh no I know better than the pump. (016)

##### Support reversing or establishing new habits and routines

Many of those who reported difficulties allowing the system to operate without (excessive) interference, also reported a history of making ‘manual micro‐tweaks’ (006) pre‐study. As well as implicating perfectionist/competitive traits, some reported ongoing difficulties reversing micromanagement practices because these had become deeply habituated (‘it's ingrained, you know, from 40 years of habit’ (005)) and, hence, were often undertaken without conscious thought:

I was just doing it [administering a corrective dose as soon as glucose started to rise] without thinking about it really, because I've been doing it for so long. (001)

Such participants expressed worries that they had not gained optimal benefit from the system because of their difficulties reversing historical and habituated micromanagement practices: ‘if I could stop tinkering, I think it would have a much better chance of it getting on with its own work’ (003). Others, in contrast, indicated needing support to develop *new* habits and routines, such as strategies to help them ‘to be mindful to try and be dosing before eating which I wasn't always good at previously’ (019).

##### Educational support

Many participants also reported educational support needs which only surfaced after they started using the system, such as dietary and/or carbohydrate counting advice; in 015's case after experiencing sharp postprandial glucose falls which had caused them to panic and turn the system off: ‘the advice I got is to be more precise about the carbs I'm putting in my pump for meals’ (015). Many also reported wanting more information about how the algorithm worked and whether, and when, they should use the exercise mode setting, after experiencing high/low glucose during or after activities such as gardening and cycling (Table 2), with 016 further suggesting that system use could be enhanced by ‘seeing a nurse who specialises in sport and exercise’.

### 3.3 Using AID systems in routine clinical care

Many participants speculated that without the glycaemic input and emotional support received from study staff, they would have struggled to thrive on the system:

I think I would've been really disappointed because it might not have been as easy to get the tweaks made or to have someone saying to me, just leave it, don't touch it and see what it does…I probably would've been frustrated…maybe would've just thought this isn't working and then sort of given up a bit. (022)

Indeed, some raised concerns that, were AID systems to be introduced widely into routine clinical care without significant investment in staff training and support provisioning, there might be high levels of system discontinuation (Table 2). Others shared their worries that older and/or less technically/numerically literate individuals would be particularly disadvantaged because they would struggle to calibrate the system without significant input from healthcare professionals (Table 2).

While participants emphasised the importance of individuals being given bespoke, tailored, one‐to‐one support, many also described being cognizant of staff shortages and resource constraints within the NHS. Hence, some suggested more pragmatic, workable solutions, such as providing technical support via short, remote data reviews (Table 2) and using a triaging system to enable staff to identify and prioritise those who would most benefit from their input (Table 2). Others highlighted benefits to offering individuals group‐based structured education programmes, such as the Dose Adjustment For Normal Eating programme (DAFNE), to help empower and upskill them to make (appropriate) adjustments to the system's profile settings (Table 2), revisit/refine carbohydrate counting skills (Table 2), and give/receive emotional support (Table 2).

## 4 DISCUSSION

This study explored the experiences, training and support needs of adults with T1D who used the Control‐IQ AID system. Mirroring findings from interview studies involving users of other AID systems,^15^, ^18^, ^20^, ^27^, ^29^ we have shown how system use can give rise to a plethora of clinical and psychosocial benefits. However, we have also shown that, for these benefits to be realised, many users would benefit from bespoke, individualised training and ongoing support from healthcare professionals, especially in the initial weeks of use comprising: glycaemic support, emotional/psychological support, support reversing or establishing new habits and educational support.

The need for initial AID training has been emphasised by others who have argued that without adequate training, users may experience safety issues and/or limited improvement in clinical outcomes.^22^ While our interviewees appreciated the training they received, many highlighted suboptimal learning experiences resulting from the use of a group‐based training format and expressed a strong preference for a more personalised/tailored approach. While one‐to‐one training should therefore be considered, this may not be feasible given the growing use of AID systems in routine clinical care. Indeed, in the UK, where AID systems have recently been made freely available to all children and young people with T1D, healthcare professionals reported pragmatic decisions to use group start‐ups due to cost, staffing, and time pressures.^30^, ^31^ Hence, when group training needs to be used, we recommend: keeping groups small to allow for more personalised learning; matching attendees according to their pace of learning, preferred ways of learning, and prior pump experience; and offering in‐person opportunities to those who prefer ‘hands on’, tactile learning approaches. To further optimise initial learning experiences, we, like others,^2^, ^22^ would also recommend developing/signposting other training resources, such as videos and simplified written materials.

Resonating with findings from other studies involving AID system use,^27^, ^29^ interviewees described healthcare professionals' role in undertaking data reviews and making recommendations for system adjustments (e.g., to correction factors, basal rates and ICRs) in the initial weeks of use as having been pivotal to successful system use. Our findings thus endorse expert recommendations that ‘Initial Device Optimization’ plans be put in place for new system users, which include tailored (possibly weekly) individualised input until the system's performance has been optimised.^2^, ^21^ In keeping with our interviewees' suggestions, this kind of support could be provided remotely (e.g., via video‐conferencing), reducing the need for in‐person visits.^2^ Additionally, consideration could be given to interviewees' suggestion of using a triaging system to allow those with greatest clinical need to be prioritised for tailored glycaemic support; indeed, such an approach has been described favourably by healthcare professionals supporting AID system use in diabetes pregnancy.^7^

In line with users' own expressed support needs, Phillip et al.^2^ note the importance of ensuring users retain/attain wider diabetes management skills (e.g., in carbohydrate counting) to help create the conditions which allow AID systems to perform optimally. Hence, it is vital that AID system users continue to be offered structured education which teaches wider diabetes self‐managements skills. To this end, programmes such as the Dose Adjustment for Normal Eating (DAFNE) programme may be especially useful as such programmes also seek to upskill and empower individuals to interpret their own glucose data and make independent adjustments to basal rates/doses, ICRs and correction factors. While use of AID systems during exercise requires further investigation,^1^ our findings indicate that many users would welcome tailored physical activity advice. This may require existing multidisciplinary teams to be expanded to include a diabetes exercise specialist.

A key assumption in clinician‐driven guidance for AID systems is that user safety, acceptance and trust can be achieved through ‘education and adjustments to ensure safe use’.^22^ While our findings suggest that educational and glycaemic support provide the necessary conditions for successful system use, these may not be sufficient. Indeed, resonating with findings from interview studies undertaken with users of other kinds of AID systems,^6^, ^14^, ^15^, ^18^, ^19^ interviewees indicated a need for emotional, psychological and/or behavioural support to help them use systems optimally. This included support to: adopt more passive diabetes self‐management roles; reverse or avoid unhelpful/habituated behaviours; sustain motivation; and, establish new habits and routines conducive to diabetes self‐management. Hence, careful consideration should be given to training and upskilling diabetes professionals in behaviour change and motivational interviewing techniques. Indeed, feedback from diabetes professionals has been very positive when such training has been received.^32^, ^33^ Additionally, to accommodate increasing use of AID systems in routine clinical care, consideration could be given to adapting existing education/training packages and structured education programmes for people with T1D to incorporate techniques from behavioural science, such as those which equip individuals with skills to identify and address unhelpful thinking patterns (e.g., perfectionism). Encouragingly, when these kinds of techniques have been incorporated into structured education, they have been shown to bring about positive changes in participants' mindsets which help facilitate long‐term improvements in their diabetes self‐management.^33^, ^34^

## 5 STRENGTHS AND LIMITATIONS

While our interviewees took part in a clinical study, they were recruited from NHS waiting lists for CGM, with no exclusion criteria applied. Consequently, we were able to access a more representative sample than typically happens in research exploring user experiences of AID systems. The initial training participants received replicated that provided within routine clinical care, which increases the applicability of our findings. However, as all of our interviewees were existing pump users, they may have experienced a less steep adjustment curve than those transitioning onto AID systems from multiple daily injection regimens. Additionally, while the Control‐IQ system offers a high level of flexibility, this system also requires high levels of input from users and healthcare professionals to get the correct settings in place.^24^ Again, this might limit the generalisability of our findings. However, reassuringly, other studies suggest that users of other kinds of AID system (e.g., CamAPS FX, Medtronic 670G/780G) would also benefit from similar psychological/behavioural, glycaemic, and educational support to that identified in this study.^6^, ^19^, ^20^, ^27^, ^29^ Future research could consider the views of individuals using AID systems in routine clinical care, including those commencing system use as pump novices. To help ensure appropriate and inclusive training and support is put in place, future research should also consider the perspectives and views of AID system users from minority ethnic groups.

## 6 CONCLUSION

AID systems are an exciting advance in diabetes treatment and can offer substantial psychosocial and clinical benefits. However, to achieve optimal gains, users would benefit from bespoke, individualised training together with ongoing glycaemic, educational, and psychological support tailored to their individual circumstances and preferred ways of learning. To help maximise the benefits of system use, consideration should also be given to adapting existing education and training packages for AID system users to incorporate techniques from behavioural science and upskilling diabetes professionals in behaviour change approaches and motivational interviewing.

## AUTHOR CONTRIBUTIONS

J.L. conceived and designed the interview study. D.R. collected the data, which was then analysed by J.L. and D.R. J.L. conceived the concept for this article. J.L. produced the first draft with input from D.R. All authors reviewed, edited, and approved the final version of the manuscript. J.L. is the guarantor of this work and, as such, had full access to all the data in the study and takes responsibility for the integrity of the data and the accuracy of the data analysis.

## FUNDING INFORMATION

This project is an investigator‐designed and driven study that was supported by a grant sought from and made available by Dexcom. The NHS research team was supported by a grant from the Chief Scientist Office through the Scottish Diabetes Research Network. The views expressed in this publication are those of the authors and Dexcom had no role in the interpretation and write‐up of the findings. For the purpose of open access, the author has applied a CC‐BY public copyright licence to any Author Accepted Manuscript version arising from this submission.

## CONFLICT OF INTEREST STATEMENT

The authors have no conflicts of interest to declare.

## CONSENT TO PARTICIPATE AND FOR PUBLICATION

All research participants provided written informed consent, including for anonymised information to be published in this article.

## Data Availability

The datasets generated and analysed in the course of this study are not publicly available due to risks to individual privacy. However, they are available, via the corresponding author, on reasonable request.
